# Reactive Arthritis Resulting From Postoperative Complications of Third Molar Extraction: A Case Report

**DOI:** 10.7759/cureus.28325

**Published:** 2022-08-23

**Authors:** Lauren Maytin, Jeffrey Morrison

**Affiliations:** 1 Behavioral Biology, Johns Hopkins University, Baltimore, USA; 2 Nutrition/Integrative Medicine, The Morrison Center, Manhattan, USA

**Keywords:** autoimmune diseases, molecular mimicry, third molar, hla-b27, reactive arthritis, case report

## Abstract

The role of bacterial infections as potential triggers for chronic rheumatic diseases is well-documented. Oral infections such as periodontitis may play a particularly important role in the development of autoimmune diseases, with the oral cavity serving as a reservoir for pathogenic bacteria. These bacteria may trigger dysregulatory immune responses through mechanisms like molecular mimicry, whereby bacterial peptides resemble self-peptides. Genetic factors may also predispose individuals to specific autoimmune diseases, most notably human leukocyte antigen B27 (HLA-B27) in the case of ankylosing spondylitis as well as other rheumatic diseases.

We present a case of a 23-year-old woman with reactive arthritis resulting from a postoperative infection. The patient presented with bilateral shoulder pain, decreased range of motion, worsening lower back pain, and mandibular pain at the site of a recent third molar extraction. Though initially diagnosed with rheumatoid arthritis, the patient experienced rapid and dramatic improvements following surgical treatment of the unresolved infection, demonstrating a causative or temporally related association between oral infections and chronic systemic autoimmune disease.

This case provides useful evidence regarding causal mechanisms for the connections between chronic autoimmune diseases and oral bacterial infection, illustrating how oral infection may serve as a causative factor for reactive arthritis. We suggest that this implicates bacteria normally present in oral microflora as a potential source of the antigens involved in triggering systemic inflammation, especially those already associated with other autoimmune diseases, such as *Klebsiella pneumoniae *in the case of ankylosing spondylitis. Based on prior publications linking self-peptides with homologies in such bacteria, we speculate on mechanisms underlying this connection, with particular attention to molecular mimicry. Clinicians should be aware of the close connection between certain rheumatic diseases (such as reactive arthritis) and bacterial infection, particularly of the oral cavity; such awareness should inform strategies for treatment and prevention of arthritic disease.

## Introduction

Autoimmune diseases constitute a significant health burden within the United States and worldwide. The overall estimated prevalence of such conditions is 4.5% and exhibits significant gender differences: 2.7% among males and 6.4% among females [1], with autoimmune diseases constituting a leading cause of death among young and middle-aged women [2]. Furthermore, the frequency of autoimmune conditions has been rising steadily in westernized society over the past few decades and across multiple disease categories: rheumatic (+7.1% per year), endocrine (+ 6.3% per year), gastrointestinal (+6.2% per year), and neurological (+3.7% per year) [3].

Despite this trend, much is still unclear about the exact mechanisms of autoimmune disorders. Genetic risk factors have been identified for certain disorders, as in the case of ankylosing spondylitis and its strong association with human leukocyte antigen B27 (HLA-B27) [4]. One prevailing theory holds that bacterial or viral infections may precipitate an immune response that leads to subsequent dysregulation. Reactive arthritis (formerly Reiter’s syndrome) specifically describes chronic, systemic inflammatory arthritis that develops in response to a bacterial infection, though associations have also been found between pathogenic bacteria and other autoimmune disorders (including ankylosing spondylitis and rheumatoid arthritis) [5].

Infections of the oral cavity in particular may serve as an important reservoir for these underlying infections, as demonstrated by the relationship between periodontal disease and autoimmune disorders described by prior studies [6-8]. This association and the case described in this report underscore the role of bacterial infection not just in localized inflammation but in systemic immune-mediated inflammatory disease.

## Case presentation

A 23-year-old woman presented to the Morrison Center with bilateral shoulder pain, significantly decreased range of motion in her left shoulder, and worsening lower back pain. The patient had a history of lower back and joint pain but developed these more severe symptoms following a surgery to remove an infected wisdom tooth which was performed two weeks prior. Before presenting to the Morrison Center, the patient was initially diagnosed with rheumatoid arthritis and sought a second opinion regarding a recommendation that she be prescribed adalimumab. Initial laboratory results revealed elevated indicators of inflammatory activity and were positive for HLA-B27 (Table 1). 

**Table 1:** Patient laboratory results CRP: C-reactive protein; ESR: erythrocyte sedimentation rate; ANA: antinuclear antibodies; -: not tested, HLA-B27: human leukocyte antigen B27

Lab review	Before Rx (June 2018)	Post Rx (Aug 2020)	Reference range
CRP (mg/dL)	10.2	-	< 1
ESR (mm/hr)	45	19	< 31
HLA-B27	pos	-	neg
ANA IgG	neg	neg	neg < 1:80

The patient was advised to continue taking meloxicam, which had been prescribed by another physician. Her symptoms of shoulder/back pain and reduced range of motion did not improve, and she began to develop worsening mandibular pain at the site of her third molar extraction. She was referred to another dentist, and it was discovered that she had developed an unresolved postoperative infection in the surgical wound; the abscess was treated with an incision and drainage procedure, and the patient received postoperative antibiotics to fully resolve the infection. There was no culture done for the drained abscess. As the patient had previously consulted a rheumatologist for testing and her joint pain was determined to be non-traumatic, no further work-up beyond bloodwork was performed to assess her joint pain and restricted range of motion.

The surgical treatment of her mandibular infection resulted in drastic improvements in the patient’s symptoms, both dental and systemic. The reported pain in her mandible, lower back, and shoulders, as well as reduced mobility of the left shoulder, all resolved completely following the procedure. This improvement was corroborated by laboratory results which indicated that levels of inflammatory markers were significantly decreased and now within the normal range (Table 1). The patient’s initial diagnosis of rheumatoid arthritis was reevaluated and determined to be reactive arthritis, a systemic inflammatory process triggered by incomplete treatment of an infection resulting from third molar extraction. No surveillance was performed for septic arthritis or infective endocarditis.

## Discussion

Third molar extractions and associated complications

Extraction of the third molars, or “wisdom teeth”, is one of the most commonly performed dental surgical procedures [9]. As of 2007, roughly 5 million Americans undergo third molar removal each year as a prophylactic measure. However, this routine surgery is also associated with a number of complications: alveolar osteitis (“dry socket”), immediate postoperative infection, postoperative bleeding, delayed-onset wound infection, and nerve damage are among the more common [10]. Reported rates for such complications vary widely from 2.6% to 30.9%; delayed-onset infections of the lower third molar site have an observed incidence of 0.7-2.2% per Figueiredo et al. [10]. The ubiquity of this procedure means that while such complications occur relatively infrequently, large numbers of patients will suffer from them each year.

Our patient’s diagnosis and pathology suggest another potential complication: infections resulting from third molar extractions may aggravate an inflammatory autoimmune response, causing acute inflammation and pain in regions beyond the immediate maxillofacial region. This relationship between delocalized inflammation and dental infections may play a role in other forms of oral disease.

Periodontitis and immune-mediated inflammatory disease

The role of bacterial infections in the etiology of systemic rheumatic diseases is well-documented, especially for such chronic conditions as rheumatoid arthritis and ankylosing spondylitis [6, 11, 12]. Periodontitis, a chronic inflammatory disease resulting from bacterial infections in the gums, has been associated with a higher risk of rheumatoid arthritis in particular and a more severe presentation of symptoms [7, 8, 13]. There is a high incidence of periodontitis in patients with rheumatoid arthritis [12], and treatment of periodontal infections has been shown to reduce symptom severity [7, 8]. Thus, chronic immune-mediated inflammatory diseases have demonstrated significant associations with infections of the mouth.

Oral infections have been implicated in other systemic arthritic diseases, acute as well as chronic. In one case study, a patient suffering from osteitis pubis experienced complete resolution of symptoms following the extraction of three teeth due to severe chronic periodontitis, accompanied by a significant decrease in C-reactive protein levels to nearly within the normal range [14]. This case displays several noteworthy similarities to the patient treated in the Morrison Center. Much like the case detailed in this report, the patient had experienced only partial improvements following treatment with anti-inflammatory medications, tested positive for HLA-B54 (which cross-reacts with HLA-B27), and was ultimately diagnosed with reactive arthritis as a result of a dental infection.

These cases provide useful evidence regarding the underlying causal mechanisms for the connections between chronic autoimmune diseases and oral bacterial infections. Though there is a clear link between oral infections and systemic inflammation, the causative factor is often difficult to identify. Periodontitis (and other infections) may cause immune-mediated inflammation through various proposed mechanisms, including metastasis of inflammatory factors, microbial toxins, or oral microbes themselves to other bodily areas [15]. Alternatively, autoimmune disorders which cause systemic bone loss may be causative of features of oral diseases like periodontitis, resulting in characteristic tooth loss, or otherwise foster poor oral hygiene through loss of dexterity. These diseases could also share no causative relationship at all and simply be a result of shared genetic risk factors interacting with environmental and behavioral features [12]. However, our case study suggests that oral infection can serve as the catalyst for systemic inflammation in at least some cases. Our patient, a previously healthy individual, developed symptoms following third molar extraction surgery, which disappeared following treatment of a post-extraction infection; this, and the clearing of inflammatory markers, draws a clear causal connection between dental infection and delocalized arthritic symptoms. This case corroborates other reports of inflammatory diseases that had a similar cause and resolution [14]. Physicians should be aware of the possibility that acute arthritic conditions may result from an underlying infection, particularly of the oral cavity, and may therefore respond better to the treatment of that infection than disease-modifying antirheumatic drugs.

HLA-B27 and ankylosing spondylitis

In addition, oral anaerobic bacteria have been implicated in the etiology of ankylosing spondylitis, an arthritic disease characterized by inflammation, pain, and stiffness of the spine. Much like rheumatoid arthritis, connections have been found between periodontitis and ankylosing spondylitis, and bacterial species involved in periodontal disease are suspected of playing a role in the development of ankylosing spondylitis in genetically susceptible individuals [6]. The presence of specific bacteria, such as *Klebsiella pneumoniae,* have been linked to inflammation in individuals with ankylosing spondylitis; other oral bacteria (including *Salmonella*, *Shigella*, *Yersinia*, and others) have been demonstrated to be causative agents in triggering reactive arthritis [4, 11]. Pathogenic oral flora can trigger or exacerbate a systemic autoimmune condition, particularly in those with genetic risk factors that predispose them to immune-mediated inflammatory disease.

Ankylosing spondylitis is notable for its strong association with HLA-B27, an antigen found on the surface of white blood cells. HLA-B27 alleles contribute much of the genetic susceptibility for ankylosing spondylitis and are frequently used as a diagnostic marker for the disease [4]. While our patient was not diagnosed with ankylosing spondylitis, her laboratory results were positive for HLA-B27, providing further evidence for the involvement of HLA-B27 in reactive arthritis. There may be other previously uninvestigated inflammatory pathways in which HLA-B27 plays a role; these may prove to be interesting avenues of study in cases of immune dysregulation.

Molecular mimicry: a possible mechanism for reactive inflammatory autoimmune diseases

Autoimmune disease occurs when the adaptive immune response, normally directed against foreign antigens, fails to properly recognize self-antigens and initiates an immune response against the body’s own tissues. Molecular mimicry describes one of the prevailing mechanisms by which bacterial infections are suspected to induce these inflammatory responses. This hypothesis suggests that certain infectious agents encode peptide sequences that bear a close similarity to those produced by the body’s own cells. When an adaptive immune response develops against pathogenic peptide sequences, it may recognize and target similar self-peptides, leading to a prolonged autoimmune response against self-antigens [16-18].

For ankylosing spondylitis and other autoimmune diseases, potential candidates for the infectious agents involved in this molecular mimicry have emerged. In ankylosing spondylitis, homologies were found between sequences of secretion proteins in *Klebsiella pneumoniae* with HLA-B27 and the enzyme pullulanase, with ankylosing spondylitis patients showing significant elevations to self-proteins for both [19]. *Klebsiella* sequences in particular have been identified as a potential agent for molecular mimicry within reactive arthritis and ankylosing spondylitis, as they were homologous with HLA-B27 amino acid sequences that showed elevated autoantibodies in patients with those diseases [20].

This association may be relevant in the case of our patient. Among other strains of enterobacteriaceae, *Klebsiella pneumoniae* has been isolated in subgingival sites of patients with chronic periodontitis [21]. Our patient is HLA-B27 positive and was diagnosed with reactive arthritis, both of which have been associated with *Klebsiella*-induced molecular mimicry. This suggests one possible mechanism by which oral infection can trigger or exacerbate systemic inflammatory disease, though further research may implicate other bacterial antigens as well.

## Conclusions

Patients with inflammatory autoimmune diseases, including rheumatoid arthritis and ankylosing spondylitis, are often prescribed disease-modifying antirheumatic drugs to slow or halt disease progression. However, bacterial infections have been increasingly recognized as a potential trigger for such immune-mediated inflammatory diseases through mechanisms such as molecular mimicry. Patients suffering from these conditions may experience a more rapid, complete, and persistent reduction of symptoms when treatments are aimed to resolve these underlying infections. The oral cavity in particular may serve as a reservoir of pathogenic bacteria that can potentially give rise to autoimmune disease, particularly as a result of surgical complications, periodontitis, and other oral infections. Additional research should be considered to further investigate the relationship between certain infections and susceptibility to developing autoimmune inflammation. This could involve recruiting patients following third molar extraction to follow prospectively for the development of rheumatic disease symptoms in comparison to controls or comparing the rates of rheumatic symptoms between those who developed an infection and those who did not. When treating patients with systemic inflammatory diseases, clinicians should be aware of this association between bacterial infection and systemic immune dysregulation and be vigilant against infections and poor oral hygiene as potential contributors to autoimmune disease.
